# Epidemiology of Rotavirus A in Nigeria: Molecular Diversity and Current Insights

**DOI:** 10.1155/2018/6513682

**Published:** 2018-10-01

**Authors:** Babatunde Olanrewaju Motayo, Adedayo Omotayo Faneye, Johnson Adekunle Adeniji

**Affiliations:** ^1^Department of Virology, University of Ibadan, Nigeria; ^2^Pathology Department, Federal Medical Centre, Idi-Aba, Abeokuta, Nigeria

## Abstract

Rotavirus induced acute gastroenteritis AGE has been a major disease burden in Nigeria, since it was first reported in 1985. Prevalence rates have increased with severe public health consequences particularly among children. The vaccine Rotarix® has been introduced and is commercially available in Nigeria. However routine rotavirus vaccination is yet to be introduced into the National Immunization Program. Molecular epidemiology of rotavirus in Nigeria has shown the presence of various genotypes, with genotype G12P[8] being the most recent introduction. There are however gaps in molecular data on rotavirus in Nigeria. We therefore reviewed molecular data on rotavirus isolated in Nigeria and also analyzed VP4 and VP7 genes of Nigerian rotavirus strains in Genbank. We have shown that there is a distinct trend in rotavirus molecular epidemiology in Nigeria, with new genotype introductions occurring after the year 2010. We also observed from our analysis the emergence of genotype G12 Lineage III as a dominant genotype. This information elucidates rotavirus molecular epidemiology in Nigeria and gives insight to the expanding landscape of rotavirus genotypes. We recommend the institution of molecular surveillance country wide, before considering the inclusion of rotavirus vaccination into the National Immunization Program in Nigeria, in other to monitor evolution of divergent or recombinant strains.

## 1. Background

Rotavirus is the leading cause of severe gastroenteritis in infants and young children worldwide; it was reported to be responsible for about 128,500 deaths in 2016, with over 70% of cases occurring in sub-Saharan Africa [1, 2]. Rotavirus causes approximately 258 million episodes of gastroenteritis requiring home care and about 24 million cases requiring medical attention [2]. Rotavirus associated mortality has drastically reduced with 528,000 deaths (range, 465,000–591,000) in 2000 to 215,000 (range, 197,000–233,000) in 2013, of which 75% occur in Africa and Asia. India and Nigeria accounted for 22% and 14%, respectively [3]. Six countries India, Nigeria, Congo, Ethiopia, China, and Pakistan account for more than half of the global mortality burden of rotavirus diarrhea [3, 4]. Rotavirus was discovered around 1973 when pictures from electron micrographs revealed small particles from thin sections of duodenal mucosa by Bishop et al. [5] and subsequently found in specimen of stool samples from children with gastroenteritis [6]. After the discovery of rotavirus, several other agents that were earlier associated with animal diarrhea diseases were later discovered to be rotaviruses from their similar morphological characteristics, such as the epizootic diarrhea of infant mice (EDIM) and simian agent 11 (SA 11) [7].

Rotavirus infects the mature villus epithelial cells of the small intestine, and infection often leads to fever, vomiting, and diarrhea in children. Dehydration and electrolyte disturbances are the major clinical sequel of rotavirus infection occurring mostly among infants. Rotavirus infection is usually localized to the intestine; however, some studies have reported antigenemia or viremia in children with rotavirus diarrhea [8, 9]. Evidence of Rotavirus infection in extra-intestinal sites, such as respiratory tract, liver, kidney, lymph nodes, and central nervous system, has also been reported [10].

The first-line preventive strategy for rotavirus infection is the development of vaccines against the virus. In the United States, the morbidity and mortality rate due to rotavirus infection have been greatly reduced following the introduction of rotavirus vaccine as recommended by the world health organization (WHO) [11]. There are currently two licensed rotavirus vaccines approved for use by the Food and drug Administration (FDA) and WHO, a bovine human recombinant pentavalent vaccine (RotaTeq®) manufactured by Merck Pharmaceuticals and a monovalent live attenuated human vaccine (Rotarix®) derived from a G1P[8] virus strain (89–12), originally developed in Cincinnati, USA, and manufactured by GlaxoSmithKline Biologicals [12]. In Nigeria, Rotarix® vaccine is available commercially but is yet to be included in the Expanded Program on Immunization (EPI).

## 2. Rotavirus Classification and Genome Organization

Rotavirus is nonenveloped RNA viruses belonging to the family Reoviridae, genus Rotavirus; other members of the family Reoviridae include genera such as Orthoreovirus, Orbivirus, Coltivirus, Cypovirus, Phytoreovirus, Aquareovirus, Oryzavirus, Seadornavirus, Idoreovirus, and Mycoreovirus [13]. The genus rotavirus is further classified according to antigenic specificities into seven serotypes A to H, based on antigenic properties of viral capsid protein VP6. This protein bears various antigenic epitopes that allow for subgroup classification (SG) among group A rotavirus. Based on this classification SGI, SGII+ SGI and II, and SG non-I and non-II have been identified based on reactivity to 2 monoclonal antibodies [14]. In 1989 a binary classification system was based on the immunologic reactivity of the two outer capsid proteins VP4 and VP7, similar to that used for influenza virus [15]. Based on this classification system there are 32 G genotypes and 47 P genotypes (https://rega.kuleuven.be/cev/viralmetagenomics/virus-classification/7th-RCWGmeeting, update of the Rega Institute, KU Leuven, Belgium).

The most recent rotavirus classification system was proposed in 2008 and involves the complete genome sequence of all 11 rotavirus gene segments with the constellation Gx-P[x]-Ix-Rx-Cx-Mx-Ax-Nx-Tx-Ex-Hx, representing genotypes of the segments VP7-VP4-VP6-VP1-VP2-VP3-NSP1-NSP2-NSP3-NSP4-NSP5/6. This classification system is based on phylogenetic analysis of all 11 gene segments and specific nucleotide cut-off identity percentages [14]. After adoption of this system a rotavirus classification working group (RCWG), which includes different scientist across various disciplines, was set up to maintain, evaluate, and monitor this system.

Rotavirus nucleic acid is composed of an 11 segmented double stranded RNA. Deproteinized rotavirus RNA is noninfectious, suggesting that they possess their own RNA-dependent RNA polymerase enzyme which they utilize to produce mRNA [16]. The rotavirus genome is about 18,500 bp in length, with each of the segments varying between 667 and 3302 bp in length [14]. The rotavirus gene sequences are A+U rich (58% to 67%), and the double stranded genome is base paired end to end and is positive sense carrying a 5′ cap with sequence: m^7^GpppG^(m)^GC [17]. Each of the positive stranded RNA segments starts with a 5′ guanidine sequence followed by a 5′ noncoding a sequence which is formed by a set of conserved sequences. This is then followed by an open reading frame (ORF) that codes for the protein product and ends with a stop codon. It is then followed by another set of noncoding sequences containing a subset of conserved terminal 3′ sequences ending with two 3′ terminal cytidines [17].

## 3. Epidemiology of Rotavirus in Nigeria

The earliest report of rotavirus induced diarrhea was conducted around communities in Oyo State, by Fagbami* et al*. [18]. Other follow-up studies revealed a high prevalence of rotavirus infection among children less than 5 years of age with acute gastroenteritis in Ibadan [19, 20]. These early studies established the presence and circulation of rotavirus among children in Nigeria but did not investigate the circulating genotypes responsible for the reported outbreaks. The earliest report demonstrating the genotype distribution of rotavirus infection among children with acute gastroenteritis in Nigeria was in 1997 [21], among children in Ibadan, Oyo State, and Maiduguri, Borno State. In this study a prevalence of 14.3% was reported; genotypes G1 and G3 were also reported. Another study by this group of researchers reported the first VP7 sequence analysis from Nigeria in 1996 [22]. These initial reports sparked interest in rotavirus research, with several workers in the following years reporting various prevalence rates of rotavirus infection among children in Nigeria.

The first major reports on the molecular epidemiology of rotavirus in Nigeria were conducted in the early to mid-1990s by the duo of Adah and Olaleye [21–23]. In these early reports, genotypes G1P[8] and G3P[6] were the predominant genotypes detected; they also reported a number of mixed genotype combinations in stools of children with gastroenteritis. The first complete VP7 nucleotide sequence of genotype G1 and G3 rotavirus isolates from Nigeria was also reported [22]. Subsequently, other workers started detecting other genotype combination such as G2P[6] and G8P[6]; mixed genotypes were also reported such as G1G8P[6], G1G9P[6], and [24, 25]. More recently Japhet* et al. *[26] detected unusual genotypes predominantly genotype G12P[8] strains and later Ayolabi* et al. *[27]. Alkali et al. [28] detected G4P[8], G4P[6], and G3P[6] strains among children from Sokoto. The most recent report on rotavirus molecular characterization reported unusual G3 strains not previously identified in Africa [29]. Another landmark report was that of Adah* et al. *[30] that showed evidence of a possible intra host recombinant G8P[1] human rotavirus strain HMG035 which was very similar to that of a bovine A5 strain from Thailand. In 2016 the first complete genome sequences of rotavirus isolates from Nigeria were reported [31]. These sequence were derived from the Bovine/Human reassortant G8P[1], HMG035 G8P[1], and NGRBg8 G8P[1]. To date these are the only completely sequenced Nigerian rotavirus isolates.

## 4. Molecular Diversity of Rotavirus VP7 (G) and VP4 [P] Genotypes in Nigeria 1994 to 2015

To investigate the molecular evolution of rotavirus genotypes from 1994 when the first rotavirus sequence was submitted to GenBank and 2016, we searched and downloaded all available Nigerian rotavirus VP4 and VP7 genes sequences in GenBank, with the help of the online data base rotavirus resource, available in the National Center for Bioinformatic Information (NCBI). Downloaded sequences were aligned using Clustal W program and phylogenetic analysis done using the neighbor joining algorithm with 1000 bootstrap replicates, using molecular evolution genetic analysis (MEGA 6.0) software, http://www.megasoftware.net. Amino acid alignment was done with Bioedit www.mbio.ncsu.edu/bioedit/. Sequence logos representing aligned amino acid sequences were generated in the WebLogo program available at https://weblogo.berkeley.edu/logo.cgi. Supplementary Tables 1 and 2 show the list of Nigeria rotavirus sequences downloaded from the National Centre for Biotechnology Information (NCBI) database, rotavirus resource for the available Nigerian rotavirus VP7 and VP4 sequences in GenBank.

**Figure 1:**
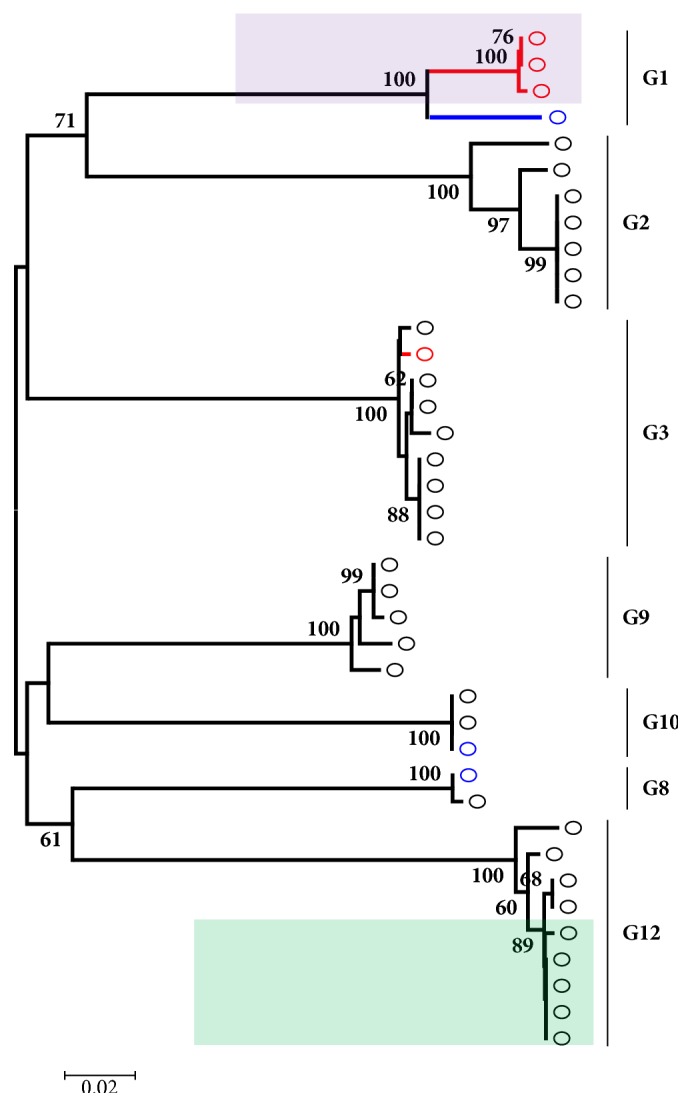
Phylogenetic analysis of the partial VP7 (G) gene sequence of Nigerian rotavirus strains from 1994 to 2015. Environmental isolates recovered from sewage effluent are shown in red; clinical isolates submitted to Genbank before the year 2000 are shown in blue. Genotype assignments are indicated by the side of the horizontal bars in the tree. The light purple shaded region shows isolates belonging to lineage II genotype G1, while the light green shaded region shows isolates belonging to lineage III genotype G12. The phylogenetic tree was constructed using neighbor joining algorithm in MEGA 6.0 with 1000 bootstrap replicates. The scale bar indicates number of substitutions per site.

**Figure 2:**
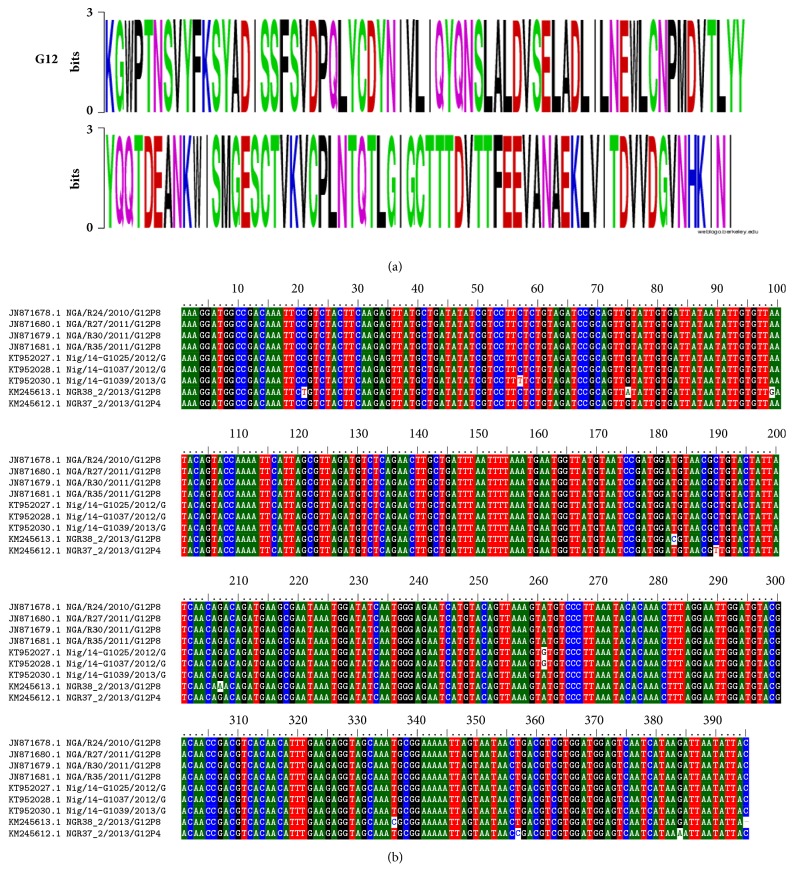
(a) Alignment of amino acid motifs from positions 112 through 243 of rotavirus group A (VP7), G12 sequences from Nigeria, showing relative abundance of individual amino acid at each position analyzed. The bit size of each amino acid is directly related to the frequency of its presence in the alignment. (b) Nucleotide sequence alignment of the 395bp region codding for the partial VP7 gene corresponding to the amino acid position 112 through 243 motifs.

From our analysis, Nigeria VP7 sequences clustered into seven genotypes, G1, G2, G3, G8, G9, G10, and G12. The older strains isolated before the year 2000 fell within genotypes G1, G8, and G10, while some of the more recent isolates, recovered after the year 2010, fell within G8, G9, and G10. Recently isolated strains from sewage effluent [32] fell within G1 and G3 as shown in Figure 1. Majority of these sequences have since been published in various journals over the years [21, 26, 30, 32]. Rotavirus G1 isolates clustered into 2 lineages with the sewage isolates falling in lineage II and the clinical isolate in lineage I as previously reported [32]. Majority of the VP7 sequences recovered between the years 2010 and 2013 belonged to genotypes G3 and G12 and were recovered in two separate outbreaks. The first outbreak took place in Ile-Ife in South Western, Nigeria, in 2011 [26], while the second outbreak took place in Enugu, Eastern Nigeria, Ile-Ife South West, Nigeria, and Maiduguri Northern Nigeria, in 2013 [29, 33]. Virtually all the G3 isolates, particularly the 2011 outbreak strains, fell within lineage III. All genotype G12 strains from 2011/2013 outbreaks fell within lineage III of genotype G12 as shown in Figure 1. There were only 10 nucleotide changes occurring within the 395bp region of the corresponding VP7 gene sequence in three isolates among nine analyzed (Figure 2(b)); this shows genetic conservation of the VP7 gene of Nigerian G12 rotavirus strains.

**Figure 3:**
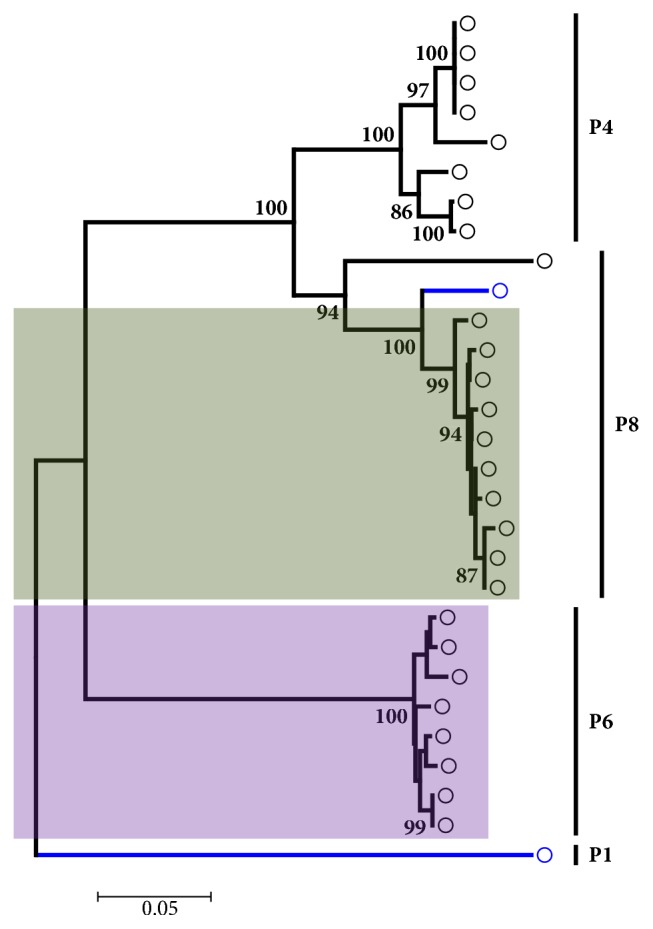
Phylogenetic analysis of the partial VP4 (P) gene sequences of Nigerian rotavirus strains from 1994 to 2015. Clinical isolates submitted to Genbank before the year 2000 are shown in blue. Genotype assignments are indicated by the side of the horizontal bars in the tree. The light grey shaded region shows isolates belonging to lineage III genotype P[8], while the light purple shaded region shows isolates belonging to lineage I genotype P[6]. The phylogenetic tree was constructed using neighbor joining algorithm in MEGA 6.0 with 1000 bootstrap replicates. The scale bar indicates number of substitutions per site.

The molecular diversity of Nigerian VP4 genotype is less extensive than it is for VP7. From our analysis VP4 was distributed into four genotypes, namely, P4, P6, P8, and P1. Virtually all the VP4 gene sequences were submitted after the year 2000, except for one HMC035 G8P[1] isolated in 1999 by Adah* et al. *[30]. Majority of the sequences analyzed were from quite recent isolates precisely between 2012 and 2013 [29, 33]. From our analysis, majority of genotype P[8] isolates fell within lineage III, while genotype P[6] isolates fell within lineage I (Figure 3); this is concordant to previous reports [29, 33]. Amino acid and nucleotide alignments of Nigerian G12 partial sequences were shown to be conserved, with complete amino acid motif conservation between analyzed G12 isolates. The above descriptions summarize VP7 and VP4 genetic diversity and phylogeny of Nigerian rotavirus isolates.

## 5. Discussion

Rotavirus disease has been established in Nigeria, causing acute gastroenteritis (AGE) with severe clinical manifestations mostly among children under five years of age [2, 34]. Rotavirus vaccine has been introduced into the commercial market for use in Nigeria but has not been included into the National Immunization Program. We have also witnessed the emergence of different genotypes including inter-species recombinants [30, 31]. One of the most comprehensive reports of rotavirus induced diarrhea was published in 2010 [25]. Aminu* et al. *[25] reported in this study a prevalence of 18% in children with diarrhea and 7% in age matched controls; genotype G1P[8] was reported to be the most abundant genotype. Other reports include a study among diarrheic children in Jos [35], which gave a prevalence of 13.8%. More recent studies have shown an increase in the prevalence of rotavirus induced diarrhea in different parts of Nigeria. For instance, a study by Iyoha* et al*. [36] reported a prevalence of rotavirus induced acute gastroenteritis of 19.2% among children in Benin City, Nigeria. Another study done in Sokoto, Northern Nigeria, among under five children also reported a high prevalence of rotavirus induced diarrhea with 25% positivity to rotavirus antigen by ELISA [37]. A study conducted among children in Ibadan in 2016 reported a prevalence of 18.5% [38]. The most recent report from Northern Nigeria recorded a prevalence rate of 32.2% of rotavirus induced gastroenteritis among children in Kaduna [39].

Molecular epidemiology of rotavirus in Nigeria has demonstrated a shift from the common globally circulating genotypes G1P[8] and G1P[6] which was reported before the turn of the new millennium [23, 27], to genotypes such as G9P[8], G12[8], G12P[6], [26–29]. Laboratory surveillance of rotavirus induced AGE in Nigeria has been very poor, with lack of diagnostic facilities in most health care institutions. This leaves a huge gap in knowledge of actual rotavirus AGE burden in the country. Most countries that have introduced rotavirus vaccination into their EPI program have instituted molecular surveillance to monitor the trend of existing genotypes and emergence of divergent as well as vaccine escape mutant strains [40, 41]. A large clinical trial follow-up for rotavirus genotype G8 spanning a period of 2 years, during the clinical trial of the Human-Bovine reassortant pentavalent vaccine Rotateq®, was conducted across 3 sites in West Africa. The study revealed that African G8 strains had a similar bovine genetic backbone with the vaccine G8 strain [41]. This information has given insight to the reason for the efficacy of the pentavalent rotavirus vaccine RotaTeq® [41]. This is just one example of how molecular epidemiology has helped in rotavirus control effort in developing countries. In Nigeria, this type of long term multisite molecular study is lacking, and majority of molecular studies conducted are short-term institutional studies often carried out by academics [23, 29, 33]. In this review we analyzed rotavirus VP7 and VP4 gene sequences isolated from 1994 to 2015, a period spanning 21 years. During this time seven VP7 genotypes were reported from Nigeria, genotypes G1, G2, G3, G8, G9, G10, and G12, with genotypes G9 and G12 being the most recent isolates [22–24, 26–29]. Environmental isolates recovered from sewage effluent were also identified in our analysis [42]. Our analysis shows a rapid emergence and dominance of genotype G12 lineage III in Nigeria. There was also specific genetic conservation within the glycoprotein gene of the isolates in this genotype, suggesting a common ancestral origin of this genotype in Nigeria. Genotype G12 has rapidly become one of the predominant genotypes in many countries [43]. The reason for its rapid spread has not yet being fully understood and could be attributed to its relatively recent introduction in countries such as Nigeria. Genotype VP4 was less diverse from our analysis as only 4 genotypes have been identified with genotype P[8] being the most predominant. There is insufficient molecular data on rotavirus in Nigeria as we could only retrieve two isolates with complete genome sequence NGRBg8 G8P[1] isolated in 1998 and HMGO35 G8P[1] isolated in 1999 [31]. This Bovine-Human reassortant isolates have very similar genetic structure, with a RVA constellation of G8P[1]-I2-R2-C2-M2-A11-N2-T6-E2-H3. Phylogenetic analysis of isolate NGRBg8 G8P[1] revealed that an interspecies transmission event must have occurred from bovine to human [31].

## 6. Conclusion

Rotavirus infection has been established to be one of the major causes of childhood diarrhea in Nigeria [20, 23, 30]. Genotypes G1P[8] and G2P[6] have been shown to predominate in Nigeria [23, 24, 29]. Recent introduction of more diverse genotypes has also been reported. New introductions of diverse genotypes continue to occur despite the availability of rotavirus vaccine Rotarix®. Genotype G12 now appears to be rapidly spreading causing outbreaks in different parts of the country [29, 33]. With these observations, there is an urgent need for the Nigerian health authorities to implement a nationwide surveillance system for monitoring rotavirus molecular epidemiology, before considering introduction of rotavirus vaccination into the expanded program on immunization (EPI) program. This will help to give necessary information on current genotypes and novel introductions as well as evolution of mutant strains to help augment current rotavirus prevention and control.

## Supplementary Materials

Supplementary Materials Supplementary tables showing Nigerian rotavirus sequences downloaded from GeneBank used in this study. Table 1: Nigerian rotavirus VP7 gene sequences used for phylogenetic analysis retrieved from gene bank. Table 2: Nigerian rotavirus VP4 gene sequences used for phylogenetic analysis retrieved from gene bank.
