# Supplementary material for: Epidemiology of Rotavirus A in Nigeria: Molecular Diversity and Current Insights
Source: J Pathog. 2018 Oct 1;2018:6513682. doi: 10.1155/2018/6513682 (PMC6188771; doi:10.1155/2018/6513682)
Supplement: Supplementary Materials — Supplementary tables showing Nigerian rotavirus sequences downloaded from GeneBank used in this study. Table 1: Nigerian rotavirus VP7 gene sequences used for phylogenetic analysis retrieved from gene bank. Table 2: Nigerian rotavirus VP4 gene sequences used for phylogenetic analysis retrieved from gene bank. [file 6513682.f1.docx]

Table 1: Nigerian rotavirus VP7 gene sequences used for phylogenetic analysis retrieved from gene bank.

| No | Name | Geneotype | Year | Assen no | Reference |
| --- | --- | --- | --- | --- | --- |
| 1 | NGA/HMG035/1999 | G8P[1] | 1999 | [LC119098](https://www.ncbi.nlm.nih.gov/nucleotide/LC119098) | Adah et al, 2001 |
| 2 | NGR/35A-396E/2014 | G1P[4] | 2014 | [KU866452](https://www.ncbi.nlm.nih.gov/nucleotide/KU866452) | Motayo et al, 2017 |
| 3 | NGR/11A-355E/2014 | G1P[4] | 2014 | [KU866451](https://www.ncbi.nlm.nih.gov/nucleotide/KU866451) | Motayo et al, 2017 |
| 4 | NGR/39A-405E/2014 | G1P[4] | 2014 | \|  \| [KU866453](https://www.ncbi.nlm.nih.gov/nucleotide/KU866453) \| \| --- \| --- \| | Motayo et al, 2017 |
| 5 | NGR/39C-400E/2014 | G3P[6] | 2014 | [KU866454](https://www.ncbi.nlm.nih.gov/nucleotide/KU866454) | Motayo et al, 2017 |
| 6 | G10 isolate 6717/2002/ARN | G10 | 2002 | [EF218662](https://www.ncbi.nlm.nih.gov/nucleotide/EF218662) |  |
| 7 | G10 isolate 6721/2000/ARN | G10 | 2000 | [EF218663](https://www.ncbi.nlm.nih.gov/nucleotide/EF218663) |  |
| 8 | NGA/R35/2011 | G12P[8] | 2011 | [JN871681](https://www.ncbi.nlm.nih.gov/nucleotide/JN871681) |  |
| 9 | NGA/R30/2011 | G12P[8] | 2011 | [JN871679](https://www.ncbi.nlm.nih.gov/nucleotide/JN871679) |  |
| 10 | NGA/R27/2011 | G12P[8] | 2011 | [JN871680](https://www.ncbi.nlm.nih.gov/nucleotide/JN871680) |  |
| 11 | NGA/R24/2010 | G12P[8] | 2010 | [JN871678](https://www.ncbi.nlm.nih.gov/nucleotide/JN871678) | Inario et al, 2015 |
| 12 | NGR/NGR86/2013 | G2P[4] | 2013 | [KM245591](https://www.ncbi.nlm.nih.gov/nucleotide/KM245591) | Inario et al, 2015 |
| 13 | NGR/NGR90/2013 | G2P[4] | 2013 | [KM245592](https://www.ncbi.nlm.nih.gov/nucleotide/KM245592) | Inario et al, 2015 |
| 14 | NGR/NGR81/2013 | G2P[4] | 2013 | \|  \| [KM245589](https://www.ncbi.nlm.nih.gov/nucleotide/KM245589) \| \| --- \| --- \| | Inario et al, 2015 |
| 15 | NGR/NGR79/2013 | G2P[4] | 2013 | [KM245588](https://www.ncbi.nlm.nih.gov/nucleotide/KM245588) | Inario et al, 2015 |
| 16 | NGR/NGR70/2013 | G3P[6] | 2013 | [KM245608](https://www.ncbi.nlm.nih.gov/nucleotide/KM245608) | Inario et al, 2015 |
| 17 | NGR/NGR75/2013 | G3P[6] | 2013 | [KM245609](https://www.ncbi.nlm.nih.gov/nucleotide/KM245609) | Inario et al, 2015 |
| 18 | NGR/NGR37_2/2013 | G12P[4] | 2013 | [KM245612](https://www.ncbi.nlm.nih.gov/nucleotide/KM245612) | Inario et al, 2015 |
| 19 | NGR/NGR56/2013 | G3P[6] | 2013 | KM245606 | Inario et al, 2015 |
| 20 | NGR/NGR51/2013 | G3P[8] | 2013 | KM245605 | Inario et al, 2015 |
| 21 | NGR38_2/2013 | G12P[8] | 2013 | KM245613 | Inario et al, 2015 |
| 22 | NGR17_2/2013 | G2P[4] | 2013 | KM245587 | Inario et al, 2015 |
| 23 | Nig/14-G1086/2013 | G9P[8] | 2013 | [KT952021](https://www.ncbi.nlm.nih.gov/nucleotide/KT952021) | Japhet et al, 2017 |
| 24 | Nig/14-G1085/2012 | G9P[8] | 2012 | [KT952023](https://www.ncbi.nlm.nih.gov/nucleotide/KT952023) | Japhet et al, 2017 |
| 25 | Nig/14-G1039/2013 | G12P[8] | 2013 | [KT952030](https://www.ncbi.nlm.nih.gov/nucleotide/KT952030) | Japhet et al, 2017 |
| 26 | Nig/14-G1037/2012 | G12P[8] | 2012 | [KT952028](https://www.ncbi.nlm.nih.gov/nucleotide/KT952028) | Japhet et al, 2017 |
| 27 | Nig/14-G1034/2013 | G9P[4] | 2013 | [KT952025](https://www.ncbi.nlm.nih.gov/nucleotide/KT952025) | Japhet et al, 2017 |
| 28 | Nig/14-G1025/2012 | G12P[8] | 2012 | [KT952027](https://www.ncbi.nlm.nih.gov/nucleotide/KT952027) | Japhet et al, 2017 |
| 29 | Nig/14-G0999/2013 | G2P[4] | 2013 | [KT952020](https://www.ncbi.nlm.nih.gov/nucleotide/KT952020) | Japhet et al, 2017 |
| 30 | Nig/14-G0990/2013 | G2P[6] | 2013 | [KT952018](https://www.ncbi.nlm.nih.gov/nucleotide/KT952018) | Japhet et al, 2017 |
| 31 | Nig/14-G1017/2013 | G9P[8] | 2013 | [KT952024](https://www.ncbi.nlm.nih.gov/nucleotide/KT952024) | Japhet et al, 2017 |
| 32 | NGR/NGR14 | G3P[8] | 2013 | [KM245596](https://www.ncbi.nlm.nih.gov/nucleotide/KM245596) | Japhet et al, 2017 |
| 33 | NGR/NGR11/2013 | G3P[6] | 2013 | [KM245595](https://www.ncbi.nlm.nih.gov/nucleotide/KM245595) | Inario et al, 2015 |
| 34 | NGR/NGR02/2013 | G3P[6] | 2013 | [KM245593](https://www.ncbi.nlm.nih.gov/nucleotide/KM245593) | Inario et al, 2015 |
| 35 | G10 isolate 6730/1999/ARN | G10 | 1999 | [EF218664](https://www.ncbi.nlm.nih.gov/nucleotide/EF218664) | Adah et al, 2001 |
| 36 | G8 VP7 protein (VP7) gene | G8 | 1998 | [AF359359](https://www.ncbi.nlm.nih.gov/nucleotide/AF359359) | Adah et al, 2001 |
| 37 | G9 VP7 protein (VP7) gene | G9 | 1998 | [AF359358](https://www.ncbi.nlm.nih.gov/nucleotide/AF359358) | Adah et al, 2001 |
| 38 | NGR/MGH066 | G1 | 1996 | Y08033 | Adah et al, 1996 |
| 39 | NGR/NGR19/2013 | G3P[8] | 2013 | [KM245597](https://www.ncbi.nlm.nih.gov/nucleotide/KM245597) | Inario et al. 2015 |

Table 2: Nigerian rotavirus VP4 gene sequences used for phylogenetic analysis retrieved from gene bank.

| No | Name | Genotype | Year | Ancess no | Ref |
| --- | --- | --- | --- | --- | --- |
| 1 | NGR/NGR02/2013/G3P[6] | P[6] | 2013 | [KM245625](https://www.ncbi.nlm.nih.gov/nucleotide/KM245625) | Inario et al, 2015 |
| 2 | NGR/NGR17_2/2013/G2P[4] | P[4] | 2013 | [KM245614](https://www.ncbi.nlm.nih.gov/nucleotide/KM245614) | Inario et al, 2015 |
| 3 | NGR/NGR24/2013/G12P[8] | P[8] | 2013 | [KM245622](https://www.ncbi.nlm.nih.gov/nucleotide/KM245622) | Inario et al, 2015 |
| 4 | NGR/NGR37_2/2013/G12P[4] | P[4] | 2013 | [KM245615](https://www.ncbi.nlm.nih.gov/nucleotide/KM245615) | Inario et al, 2015 |
| 5 | NGR/NGR38_2/2013/G12P[8] | P[8] | 2013 | [KM245623](https://www.ncbi.nlm.nih.gov/nucleotide/KM245623) | Inario et al, 2015 |
| 6 | NGR/NGR42/2013/G3P[6] | P[6] | 2013 | [KM245626](https://www.ncbi.nlm.nih.gov/nucleotide/KM245626) | Inario et al, 2015 |
| 7 | NGR/NGR49/2013/G3P[6] | P[6] | 2013 | [KM245627](https://www.ncbi.nlm.nih.gov/nucleotide/KM245627) | Inario et al, 2015 |
| 8 | NGR/NGR58/2013/G3P[6] | P[6] | 2013 | [KM245628](https://www.ncbi.nlm.nih.gov/nucleotide/KM245628) | Inario et al, 2015 |
| 9 | NGR/NGR70/2013/G3P[6] | P[6] | 2013 | [KM245629](https://www.ncbi.nlm.nih.gov/nucleotide/KM245629) | Inario et al, 2015 |
| 10 | NGR/NGR78/2013/G3P[4] | P[4] | 2013 | [KM245616](https://www.ncbi.nlm.nih.gov/nucleotide/KM245616) | Inario et al, 2015 |
| 11 | NGR/NGR81/2013/G2P[4] | P[4] | 2013 | [KM245617](https://www.ncbi.nlm.nih.gov/nucleotide/KM245617) | Inario et al, 2015 |
| 12 | NGR/NGR83/2013/G2P[4] | P[4] | 2013 | [KM245618](https://www.ncbi.nlm.nih.gov/nucleotide/KM245618) | Inario et al, 2015 |
| 13 | NGR/NGR86/2013/G2P[4] | P[4] | 2013 | [KM245619](https://www.ncbi.nlm.nih.gov/nucleotide/KM245619) | Inario et al, 2015 |
| 14 | NGA/HMG035/1999/G8P[1] | P[1] | 1999 | [LC119096](https://www.ncbi.nlm.nih.gov/nucleotide/LC119096) | Adah et al, 2001 |
| 15 | G10 strain 6717/2002/ARN | P[8] | 2002 | [GU997035](https://www.ncbi.nlm.nih.gov/nucleotide/GU997035) |  |
| 16 | Nig/14-G1035/2013/G12P[8] | P[8] | 2013 | [KT952039](https://www.ncbi.nlm.nih.gov/nucleotide/KT952039) | Japhet et al, 2017 |
| 17 | Nig/14-G1076/2013/G12P[8 | P[8] | 2013 | [KT952041](https://www.ncbi.nlm.nih.gov/nucleotide/KT952041) | Japhet et al, 2017 |
| 18 | Nig/14-G1054/2013/G12P[8] | P[8] | 2013 | [KT952042](https://www.ncbi.nlm.nih.gov/nucleotide/KT952042) | Japhet et al, 2017 |
| 19 | Nig/14-G1085/2012/G9P[8] | P[8] | 2012 | [KT952037](https://www.ncbi.nlm.nih.gov/nucleotide/KT952037) | Japhet et al, 2017 |
| 20 | Nig/14-G0992/2012/G9P[8] | P[8] | 2012 | [KT952036](https://www.ncbi.nlm.nih.gov/nucleotide/KT952036) | Japhet et al, 2017 |
| 21 | Nig/14-G0999/2013/G2P[4] | P[4] | 2013 | [KT952046](https://www.ncbi.nlm.nih.gov/nucleotide/KT952046) | Japhet et al, 2017 |
| 22 | Nig/14-G1034/2013/G9P[4] | P[4] | 2013 | [KT952047](https://www.ncbi.nlm.nih.gov/nucleotide/KT952047) | Japhet et al, 2017 |
| 23 | Nig/14-G1042/2012/G12P[8] | P[8] | 2012 | [KT952040](https://www.ncbi.nlm.nih.gov/nucleotide/KT952040) | Japhet et al, 2017 |
| 24 | Nig/14-G1086/2012/G9P[8] | P[8] | 2012 | [KT952044](https://www.ncbi.nlm.nih.gov/nucleotide/KT952044) | Japhet et al, 2017 |
| 25 | Nig/14-G1048/2013/G12P[8] | P[8] | 2013 | [KT952043](https://www.ncbi.nlm.nih.gov/nucleotide/KT952043) | Japhet et al, 2017 |
| 26 | Nig/14-G1048/2013/G2P[6] | P[6] | 2013 | [KT952034](https://www.ncbi.nlm.nih.gov/nucleotide/KT952034) | Japhet et al, 2017 |
| 27 | Nig/14-G1089/2012/G3P[6] | P[6] | 2012 | [KT952035](https://www.ncbi.nlm.nih.gov/nucleotide/KT952035) | Japhet et al, 2017 |
| 28 | Nig/14-G0990/2013/G2P[6] | P[6] | 2013 | [KT952033](https://www.ncbi.nlm.nih.gov/nucleotide/KT952033) | Japhet et al, 2017 |
| 29 | Nig/14-G1008/2013/G12P[8] | P[8] | 2013 | [KT952038](https://www.ncbi.nlm.nih.gov/nucleotide/KT952038) | Japhet et al, 2017 |
